# Mechanisms of Bone Impairment in Sickle Bone Disease

**DOI:** 10.3390/ijerph18041832

**Published:** 2021-02-13

**Authors:** Paola Giordano, Flavia Urbano, Giuseppe Lassandro, Maria Felicia Faienza

**Affiliations:** Paediatric Unit, Department of Biomedical Sciences and Human Oncology, University of Bari “A. Moro”, 70124 Bari, Italy; flaviaurbano84@gmail.com (F.U.); giuseppelassandro@live.com (G.L.)

**Keywords:** sickle cell disease, bone, metabolism, growth, endocrine complications

## Abstract

Sickle bone disease (SBD) is a chronic and invalidating complication of Sickle cell disease (SCD), a multisystem autosomal recessive genetic disorder affecting millions of people worldwide. Mechanisms involved in SBD are not completely known, especially in pediatric age. Among the hypothesized pathogenetic mechanisms underlying SBD are bone marrow compensatory hyperplasia and bone ischemic damage, both secondary to vaso-occlusive crisis (VOC), which leads to cell sickling, thus worsening local hypoxia with a negative impact on osteoblast recruitment. Furthermore, the hypoxia is a strong activator of erythropoietin, which in turn stimulates osteoclast precursors and induces bone loss. Hemolysis and iron overload due to a chronic transfusion regimen could also contribute to the onset of bone complications. Vitamin D deficiency, which is frequently seen in SCD subjects, may worsen SBD by increasing the resorptive state that is responsible for low bone mineral density, acute/chronic bone pain, and high fracture risk. An imbalance between osteoblasts and osteoclasts, with a relative decrease of osteoblast recruitment and activity, is a further possible mechanism responsible for the impairment of bone health in SCD. Moreover, delayed pubertal growth spurt and low peak bone mass may explain the high incidence of fracture in SCD adolescents. The aim of this review was to focus on the pathogenesis of SBD, updating the studies on biochemical, instrumental, and biological markers of bone metabolism. We also evaluated the growth development and endocrine complications in subjects affected with SCD.

## 1. Introduction

**Figure 1:**
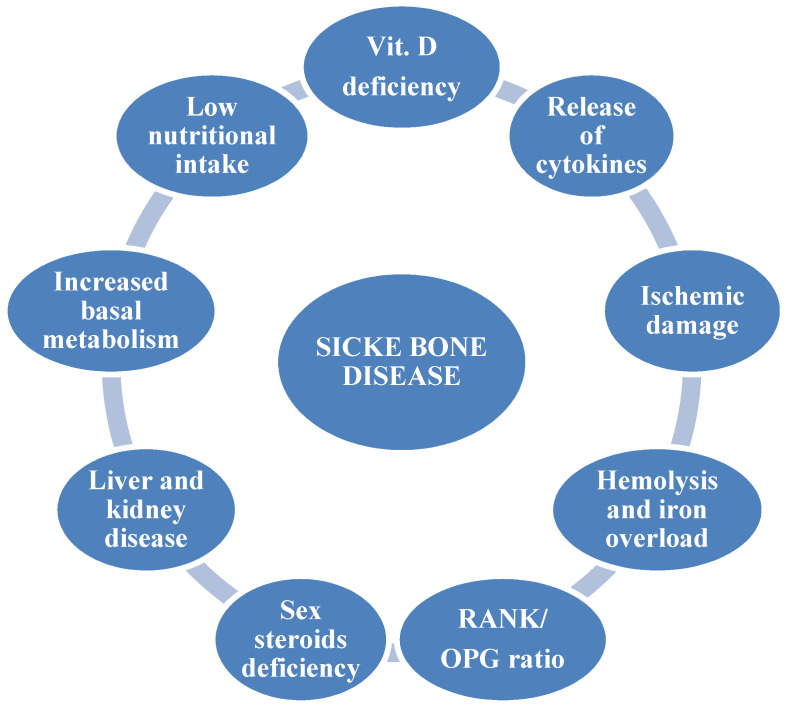
Pathogenetic factors of sickle bone disease.

Sickle cell disease (SCD) is an inherited autosomal recessive disorder affecting millions of people worldwide [1]. According to the Global Burden of Disease Study [2], 3.2 million people are affected with SCD, 43 million people are carriers, and 176 thousand people die because of SCD-related complications each year. Sickle cell anemia (SCA) is the most common form, accounting for 70% of cases of SCD in African Americans [3]. A single amino acid substitution in the β-globin chain results in the production of the characteristic hemoglobin S (HbS) which, when deoxygenated, causes red blood cells to take on the typical sickle shape. Clinical manifestations of SCD are variable and affect multiple organs, generally causing a shorter life expectancy. The major cause of morbidity and acute care in this population is represented by acute vaso-occlusive crisis (VOC) due to microvascular occlusion, which leads to increased inflammation and tissue ischemic damage [4]. Bone involvement is common, due to both acute painful VOC and progressive disabilities caused by avascular necrosis. Other acute bone complications in SCD patients are represented by osteomyelitis [5], orbital bone infarction [6], dental problems [7], and bone marrow necrosis. Another severe complication is represented by fat embolism syndrome (FES) consequent to long bone fractures and orthopedic surgery, and characterized by respiratory failure, neurological involvement, skin rashes, and thrombocytopenia [8]. Instead, non-traumatic FES is due to extensive bone marrow necrosis, and is associated with high mortality rates and severe neurological sequelae. The pathophysiological mechanism is unknown, although it exclusively affects patients with mild forms of the disease, often in the context of parvovirus B19 infection [9]. In addition to acute bone involvement, sickle bone disease (SBD) represents a chronic and invalidating complication of SCD. About 80% of SCD young adults have low bone mineral density (BMD), osteopenia, osteoporosis, increased risk of fractures, vertebral collapse, and bone pain [10,11,12]. The reduced BMD in SCD subjects is independent of risk factors typical for the general population such as age, sex, and menopause. Bone loss could partially depend on different factors such as vitamin D insufficiency, low physical activity, malnutrition, and release of inflammatory cytokines (Figure 1) [13]. However, the mechanisms involved in the pathogenesis of SBD are not completely known, especially in pediatric age, and there are no targeted therapies to prevent or treat bone impairment in this population. The bone marrow compensatory hyperplasia and bone ischemic damage secondary to VOC lead to cell sickling, worsening the local hypoxia with a negative impact on osteoblast recruitment. Recent studies have demonstrated that the immediate response to hypoxia is the activation of hypoxia-inducible factor (HIF), which induces the transcription of genes and factors modulating oxygen homeostasis, like erythropoietin, which stimulates osteoclast precursors and induces bone loss [14,15].

This results in cortical thinning and increased bone fragility. In addition, hemolysis and iron overload due to a chronic transfusion regimen also contribute to the onset of bone complications [16]. Iron is an essential cofactor for hydroxylation of prolyl and lysin residues of procollagen and contributes to vitamin D metabolism through the cytochromes P450 [17].

Recently, vitamin D deficiency has emerged as a public health concern due to its skeletal and extra-skeletal effects [18]. Children with SCD show a high prevalence of this condition, ranging from 56% to 96% [19]. The exact reason for this vitamin deficiency is unknown, but several etiologies, such as dark skin color, limited sunlight exposure, poor nutritional intake, low nutrient absorption, and renal dysfunction have been hypothesized [20,21]. Moreover, SCD subjects have an increased basal metabolism due to the continuous production of red blood cells to compensate the shortened red blood cell survival [22]. Vitamin D deficiency worsens SBD through an increase of the resorptive state that is responsible for low BMD, acute/chronic bone pain, and high fracture risk [19,23]. Additionally, liver and kidney disease related to SCD may negatively contribute to bone homeostasis.

An imbalance between osteoblasts (OBs), the bone forming cells, and osteoclasts (OCs), the bone reabsorbing cells, with a relative decrease of osteoblast recruitment and activity, has been hypothesized as a possible mechanism responsible for the impairment of bone homeostasis in SCD. In particular, the RANK/RANKL/osteoprotegerin (OPG) axis, which controls osteoclastogenesis, could have a role in the altered bone health in SCD subjects [24].

In addition, the deficit of sex steroids, in particular of estradiol both in males and females, could contribute to bone damage [25], as demonstrated by bone impairment in disorders of sexual development [26]. In contrast with these findings, high-BMD values in SCD patients, particularly in those with S/β-thalassemia genotypes, have been observed [27]. Osteosclerosis is due to multiple infarctions consequent to VOC, leading to reduced osteoclast activity and increased BMD [27].

Impaired growth is a known complication in SCD in children, in part due to marrow hyperplasia [28]. In addition, vitamin A and B6 deficiencies in SCD do not seem to be related to malnutrition per se, but to a high resting energy expenditure [29]. Furthermore, SCD children show a high prevalence of metabolic and endocrine alterations, thus achieving good clinical control is an important goal in order to avoid severe growth impairment and related complications in SCD patients.

## 2. Objective

In this review, we focused on the pathogenesis of SBD, updating the studies on biochemical, instrumental, and biological markers of bone metabolism. We also evaluated data on growth development and endocrine complications in subjects affected with SCD.

## 3. Methods

This review was conducted according to the guidelines outlined in Preferred Reporting Items for Systematic Reviews and Meta-Analyses: The (PRISMA) Statement [30].

### 3.1. Eligibility Criteria

Manuscripts considered eligible for this review included only: i. original published articles, and ii. observational or experimental studies. We found 27 papers that met these criteria.

### 3.2. Information Sources and Search Strategy

The Medline bibliographic database of the National Library of Medicine, accessed through the National Institutes of Health’s PubMed online resource, was searched for articles to include in this review. The primary search included the following key words: “sickle cell disease” AND “bone” AND “metabolism” AND “biochemical” AND “instrumental” AND “biological” AND “growth” AND “endocrine complications”. Additionally, articles referenced by those identified in this search were reviewed for relevance.

### 3.3. Study Selection

Articles were reviewed to determine if bone metabolism was assessed in the study population. Experimental studies were included only if the authors assessed markers of bone metabolism at baseline or before any treatment began.

### 3.4. Data Collection Process and Data Items

For each study that was considered eligible, the following data were collected: number and age of subjects, presence or lack of a comparison group, clinical parameters, biomarkers of bone turnover, and prevalence of metabolic or endocrine complications.

## 4. Results

Bone mass can be assessed through different methods that include the analysis of biochemical, instrumental, and biological parameters. Biomarkers of bone remodeling are classified according to their function, including markers of bone formation such as bone-specific alkaline phosphatase, osteocalcin, and C-terminal propeptide of type I collagen (C1CP), whose roles are to stimulate the deposition of new bone; and markers of bone resorption that are involved in bone remodeling, such as cross-linked deoxypyridinoline (DPD), N-terminal propeptide of type I procollagen (P1NP), cross-linked N-terminal telopeptide of type I collagen (NTX), and C terminal telopeptide of type I collagen (CTX). The main instrumental tool for the evaluation of BMD, bone area (BA), and bone mineral content (BMC) is dual X-ray absorptiometry (DEXA). Finally, the assessment of bone remodeling includes the evaluation of two pathways: the RANK/RANKL/OPG and Wnt/β-catenin pathways, which play a key role in the control of osteoclastogenesis and osteoblastogenesis, respectively.

### 4.1. Biochemical Studies

A comparison study by Fung et al. [31] reported higher levels of osteocalcin and bone-specific alkaline phosphatase, and lower levels of DPD in SCA children than healthy controls, regardless of sex and pubertal stage. However, males presented a more severe growth failure than females. Bone-specific alkaline phosphatase and osteocalcin were positively correlated with height velocity, while there was no correlation between these biomarkers and bone mass assessed by DEXA. In a cross-sectional case–control study, Mokhtar et al. [32] investigated the relationship between bone resorption, vascular complications, and BMD assessed by DEXA in a cohort of SCD children and adolescents compared to healthy controls. SCD patients had a significantly lower BMD Z-scores and higher levels of alkaline phosphatase and tartrate-resistant acid phosphatase 5b (TRACP 5b, a specific marker of osteoclast activity) than healthy controls. Furthermore, high TRACP 5b levels were associated with severe VOC, as patients treated with hydroxyurea or on chelation therapy had lower TRACP 5b levels than untreated patients. In another case–control study by Nouraie et al. [33], TRACP 5b levels were significantly higher in SCD adult patients than controls, regardless of age, sex, body mass index (BMI), and disease severity, and they strongly correlated with alkaline phosphatase. TRACP 5b levels also correlated with the proinflammatory mediators IL-6, IL-8, and endothelin-1, suggesting a potential role of inflammation in promoting osteoclast activity in SCD [34]. Furthermore, iron overload could potentially trigger osteoporosis by reducing osteoblast activity and upregulating the *TRACP5b* gene, as demonstrated in vitro by the inhibition of bone resorption after chelation therapy [32]. Bone is the main source of alkaline phosphatase, which increases during VOC due to abnormal osteoclast activity. This suggests that delayed growth and bone destruction, secondary to sickling and altered calcium metabolism, contribute to the increase of alkaline phosphatase and urinary hydroxyproline levels, leading to bone damage in SCD [35]. In an experimental study on 20 SCD adults, Bolarin et al. [36] demonstrated high levels of urinary NTX and urinary DPD in patients with both SCD and bone complications. Vitamin D deficiency is frequent among patients with SCD, with a prevalence ranging from 56% to 96% [19]. The etiology is multifactorial: dark skin color, limited sunlight exposure, poor nutritional intake and renal dysfunction are the most important factors [20,21]. A retrospective study of 53 SCD children showed a correlation between chronic pain and lower 25-OHD levels [37]. Another cross-sectional study of 95 SCD children found an association between low 25-OH vitamin D levels and acute pain [38]. A cross-sectional study by Han et al. [39] evaluated clinical and genetic variables associated with vitamin D deficiency in a cohort of 335 adult African American SCD patients. Vitamin D deficiency was found in 65% of SCD patients, and 25-OH vitamin D levels positively correlated with older age and vitamin D supplementation. Furthermore, among key genes involved in vitamin D metabolism, *CYP3A4* and *SLC6A5* expression significantly correlated with lower 25-OH vitamin D levels, while *DBP* and *VDR* expression correlated with higher levels [39]. The *SLC6A5* gene encodes glycine transporter 2 (*GlyT2*), a protein involved in the neuronal pain pathway, while *GC* and *VDR* are involved in vitamin D metabolism. In the sensory system, glycinergic neurons control the excitability of pain transmission pathways from the periphery to the brain. Chronic pain is associated with the loss of this inhibitory mechanism. GlyT2 inhibitors have been shown to restore inhibitory glycinergic function, thus providing an analgesic effect [40]. Further research is needed for the development of drugs which interfere with the glycinergic system. Furthermore, it will be necessary to better characterize the clinical benefits of routine vitamin D supplementation.

There are few studies on the impact of vitamin D deficiency on bone metabolism in SCD subjects. In a prospective study by Arlet et al. [23] conducted with 56 adult patients, vitamin D deficiency was detected in 75% of patients. Among them, 95% had secondary hyperparathyroidism due to vitamin D insufficiency and low calcium intake. History of fractures was documented in 30% patients, and osteopenia and osteoporosis in 40%. In a recent study by Gregoire-Pelchat et al. [41], the use of a high-dose vitamin D bolus combined with daily 1000 IU vitamin D3 was more effective in raising 25-OH vitamin D levels than only daily supplementation in SCD children. Further studies are needed to determine if this approach is safe and improves outcomes in children with SCD.

### 4.2. Instrumental Studies

The main instrumental tool for the evaluation of BMD, BA, and BMC is the DEXA [42]. WHO criteria are used for defining normal (T-score > −1 SD), osteopenia (T-score between −2.5 SD and −1 SD), and osteoporosis (T-score < −2.5 SD) [43]. In addition, thoracic and lumbar spine X-rays in lateral projection recognize vertebral fractures (typical “fish-shaped” vertebral bodies) which are classified according to Genant grades based on height reduction [44]. In a cohort study conducted on 71 SCD adults [45], a low prevalence of osteoporosis (ranging between 7% and 18%) based on BMD assessed by DEXA was found, despite a high prevalence of vertebral fractures and skeletal deformities, especially in patients with a more severe hemolytic phenotype. A T-score corresponding to a −1.4 standard deviation score (SDS) corresponded to the cut-off to identify the presence of fractures in patients with SCD. Thus, spine radiograph was shown to be a more powerful tool than BMD assessment for diagnosis and follow-up of SBD. Miller et al. [10] found a high prevalence of low BMD in different sites, particularly in the lumbar spine, and a correlation with male sex and BMI in adults with SCA. They also found accelerated bone turnover, documented by higher urinary NTX levels, in patients with low BMD, while no significant difference was found in osteocalcin levels. Patients with low BMD had increased disease severity and a higher risk of vertebral and hip fracture than controls, although not statistically significant. A study by Chapelon [46] assessed the prevalence of low BMD by DEXA in children with SCD. Mean lumbar spine BMD Z-scores were significantly lower in girls than boys in the prepubertal subgroup, and worsened in boys after pubertal development. Low BMC was not associated with any marker of bone and calcium metabolism, although low 25 OH vitamin D levels and urinary calcium excretion were found in most patients, and high PTH or osteocalcin levels were found especially in females. Low BMD was not associated with any anthropometric parameter, nor any marker of disease severity (number of hospitalizations, blood transfusion, VOC, and Hb level) in girls, while it negatively correlated with VOC and hospitalizations in boys. In a case–control study by Buison et al. [47], BMC and BA assessed by DXA correlated with clinical and hematologic parameters. SCD children had a significantly lower BMC and BA, adjusted for growth, pubertal delay, and reduced lean mass, than healthy children. Impairment in bone maturation was more common in male adolescents and correlated with disease severity and delayed bone age. Thus, SCD children may be at high risk of fractures and suboptimal peak bone mass.

### 4.3. Biological Studies

Bone remodeling is an active process due to the balanced activity of OCs, bone-resorbing cells, and OBs, bone forming cells, which work sequentially throughout life [48]. In healthy subjects, bone formation predominantly occurs in the first two decades of life, until the achievement of peak bone mass. Thereafter, the bone mass remains stable for approximately 20 years, until resorption begins to overcome bone formation, resulting in age-related bone loss. Osteoporosis is a common skeletal disorder characterized by altered bone strength that predisposes patients to an increased risk of fracture [49]. Among the secondary forms of osteoporosis, hematological diseases exert an important role, given the close relationships between bone and bone marrow. Bone cells interact with hematopoietic cells to maintain erythropoiesis and myelopoiesis, as demonstrated in animal models [50]. Furthermore, the effects of hematological diseases on bone are also due to the action of pathways that control the balance between osteoclast and osteoblast activity. The RANK/RANKL/ OPG and Wnt/β-catenin pathways play a key role in the control of osteoclastogenesis and osteoblastogenesis, respectively. The altered bone remodeling associated with some inherited and acquired pediatric diseases is due to an impairment of these pathways [51].

Voskaridou et al. [24] focused on the role of the RANKL/OPG axis in the pathophysiology of SBD in 52 adults with HbS/β-thalassemia. They found high OPG levels and amplified erythropoietic activity, which indicated a strong correlation among markers of bone resorption in these patients. These findings suggest that marrow hyperplasia may trigger the development of osteopenia/osteoporosis, but compensatory increase in OPG and decrease in RANKL may protect young patients from bone impairment. A recent study by Tombak et al. [52], performed on 58 adults with HbS/β-thalassemia and 52 adults with the β-thalassemia trait, demonstrated that the β-thalassemia trait alone is not a risk factor for osteopenia/osteoporosis. Furthermore, osteoporosis did not develop in premenopausal women and men younger than 50 years with HbS/β-thalassemia, but instead developed with age, as demonstrated by a compensatory decrease of osteoclastic cytokine, sRANKL, with a compensatory increase of the osteoblastic cytokine OPG.

Dalle Carbonare et al. [53] reported that osteoclast activation combined with suppressed osteoblast function contributed to the development of SBD in a mouse model under normoxia. Bone loss was demonstrated by an increased expression of RANKL, while bone synthesis was impaired, as indicated by a reduction of osteoblastic colony forming units and downregulation of the osteogenic markers RUNX2 and SPARC. Repeated hypoxia/reperfusion stress increased bone turnover by osteoclast recruitment and suppression of RUNX2 and SPARC. The administration of zoledronic acid before the induction of recurrent hypoxia/reperfusion stress prevented bone impairment.

Recently, an accumulation of aged neutrophils in the bone marrow of SCD mice, which contribute to impaired osteoblast function, has been demonstrated [54]. Antibiotic-treated SCD mice showed a partial rescue of osteoblast function when neutrophil aging in the bone marrow was decreased.

## 5. Growth Pattern and Endocrine Complications

In recent decades, survival rates among children with SCD have increased, due to an earlier diagnosis and a better quality of care. Therefore, the incidence of long-term complications such as stunted growth as well as metabolic and endocrine disorders is increasing in the SCD population. The underlying mechanism of growth failure and delayed bone maturation is very complex, and probably influenced by many variables such as hematologic and cardiovascular status, socio-economic factors, endocrine and metabolic function, and nutritional status. Growth seems to be more affected in subjects with the HbSS genotype than in subjects with the HbSC genotype [55]. Moreover, patients treated with hydroxyurea for more than one year show lower BMI-SDS and sitting height/height ratio, probably because of a more severe phenotype [55]. More studies are needed to demonstrate that the use of hydroxyurea can both improve clinical outcomes for SCD and positively influence growth and development by decreasing the risk of iron overload due to a chronic transfusion regimen.

As regards endocrine complications, in a cross-sectional clinical trial on 52 Italian children and adolescents with steady-state SCD, a high prevalence of endocrine alterations, corresponding to 92% of SCD patients, was found [55]. Consistent with previous studies [56], it was reported that vitamin D deficiency was the most common endocrine complication in these patients, followed by insulin resistance, growth hormone deficiency, hypogonadism, and hypothyroidism. Vitamin D levels negatively correlated with clinical indicators of SCD severity. On the contrary, a positive correlation was found between clinical parameters of disease severity and anthropometric parameters: children with milder SCD had higher height, weight, and BMI than those with severe SCD, regardless of age and gender. Moreover, patients who needed longer treatment with hydroxyurea had lower BMI and height than those with better control of the disease [55]. In a prospective longitudinal comparative study, Fung et al. found that the significant predictors of fractures in SCD patients were age, male gender, hypothyroidism, and hypogonadism. Regardless of gender, and despite transfusion and iron chelation therapy, patients with hypogonadism were at high risk of fractures due to low bone mass and vitamin D deficiency [31].

## 6. Discussion

Bone is one of the main targets of SCD. Furthermore, children with SCD experience poor growth, delayed bone maturation, and endocrine complications. There are few and controversial data about the pathogenesis of bone impairment in SCD. Most studies, performed predominantly in adults, reported an increase of bone resorption markers [24,32,33], supporting the hypothesis that multiple VOCs can stimulate bone resorption in SCD subjects. On the other hand, other studies have shown a decrease of osteocalcin and IGF-1 levels, suggesting impaired bone formation as an underlying mechanism for BMD deficiency [57,58]. Several factors can contribute to reduced BMD in SCD children, such as low dietary calcium and vitamin D intake, confirmed by low serum 25 OH vitamin D levels [20]. Vitamin D deficiency is the most common nutritional deficit in SCD subjects [23,59], and it could be determined by an imbalance between inadequate intestinal absorption and increased metabolic demand due to high red blood cell turnover [22,60]. Moreover, patients with SCD have renal impairment and, consequently, an inability to convert vitamin D to its active form. Finally, vitamin D binding protein decreases in inflammatory states like SCD [61]. A study of trends in vitamin D status found that the prevalence of normal levels (>30 ng/mL) in African American people aged 12 or older was only 3%, and severe deficiency/insufficiency ranged from 9% to 29% [62]. A more recent report found that 81% of African American adults were deficient (<20 ng/mL) compared to 28% of Caucasians [63]. Other studies have reported a very high prevalence of low vitamin D levels in adults with SCD [23,64]. Moreover, vitamin D levels have been negatively correlated with clinical indicators of SCD severity.

Gender and pubertal status influence bone mineralization and calcium metabolism [10]. Female subjects show a BMD Z-score significantly lower than males in the prepubertal age, whereas it worsens after pubertal development in males. Estrogens are essential for the maintenance of bone health, as demonstrated by bone impairment in syndromes with gonadal dysfunction, such as Turner syndrome, as well as in other disorders of sexual development [65,66]. Low BMD is not associated with any anthropometric parameters, nor with any markers of disease severity in SCD subjects—except in males, who show a low BMD that correlates with the number of VOCs and hospitalizations, but not with transfusions [10]. SCD children often experience growth failure and pubertal delay. Growth patterns appear to be less compromised in children with milder SCD, regardless of age and gender. A high prevalence of endocrine complications has been observed in SCD children [55], such as in subjects affected by diseases associated with a low grade of inflammation [67]. An impairment of the growth hormone (GH)–IGF-1–IGFBP3 axis has been demonstrated in SCD subjects [55,68]. Thus, growth hormone deficiency, hypothyroidism, hypogonadism, and insulin resistance are common in SCD patients [68,69].

## 7. Conclusions

From our review of the literature, osteoclast resorptive function, rather than osteoblast dysfunction, appears to play the major role in the onset of SBD. Moreover, delayed pubertal growth spurt and low peak of bone mass may explain the high incidence of fracture in SCD adolescents.

There are few studies about the pathophysiology of endocrine disorders in SCD patients. They are likely more related to VOC and ischemic events rather than iron overload, and their prevalence varies according to treatment, as well as cultural and socioeconomic factors. Interestingly, treated patients show improvement in clinical outcomes and increased survival rate over time, suggesting that good clinical control of the disease can have a positive impact on growth and metabolic and endocrine function.

Additionally, screening for oral and perioral infections and dental health should become part of health promotion programs in patients with SCD.

Further studies are needed which investigate the early detection of osteopenia/osteoporosis in SCD patients, as well as targeted therapy to reduce bone complications and improve the outcome of the disease.
